# The development and testing of a brief (‘gist-based’) supplementary colorectal cancer screening information leaflet^☆^

**DOI:** 10.1016/j.pec.2013.08.013

**Published:** 2013-12

**Authors:** Samuel G. Smith, Michael S. Wolf, Austin Obichere, Rosalind Raine, Jane Wardle, Christian von Wagner

**Affiliations:** a Cancer Research UK Health Behaviour Research Centre, Department of Epidemiology and Public Health, UCL, London, UK; b Health Literacy and Learning Program, Division of General Internal Medicine, Feinberg School of Medicine at Northwestern University, Chicago, USA; c Colorectal Unit, Department of Surgery, University College London Hospitals, London, UK; d Department of Applied Health Research, UCL, London, UK

**Keywords:** User-testing, Colorectal cancer, Screening, Fuzzy-trace theory, Health literacy, Information design, Numeracy

## Abstract

**Objective:**

To design and user-test a ‘gist-based’ colorectal cancer screening information leaflet, which promotes comprehension of the screening offer.

**Methods:**

Twenty-eight individuals approaching screening age were recruited from organisations in deprived areas of England. Using a between-subjects design, we tested iterations of a newly-designed gist-based information leaflet. Participants read the leaflet and answered 8 ‘true’ or ‘false’ comprehension statements. For the leaflet to be considered fit-for-purpose, all statements had to be answered correctly by at least 80% of participants in each round. Alterations were made if this threshold was not met and additional rounds of testing were undertaken.

**Results:**

At round 1, answers to 2/8 statements did not meet the threshold. After changes, answers in round 2 did not reach the threshold for 1/8 statements. In round 3, all answers were adequate and the leaflet was deemed fit-for-purpose. Qualitative data offered solutions such as language and layout changes which led to improved comprehension of the leaflet.

**Conclusion:**

User-testing substantially improved the design and subsequent comprehensibility of a theory-driven gist-based colorectal cancer screening information leaflet.

**Practical implications:**

This leaflet will be evaluated as part of a large national randomised controlled trial designed to reduce socioeconomic inequalities in colorectal cancer screening participation.

## 1 Introduction

### 1.1 Background

Faecal occult blood (FOB) testing is a common method of screening for colorectal cancer (CRC) [1]. Large organised programmes, such as the English CRC screening programme rely on written health communication materials to inform the public about the test. Conceptual frameworks suggest the ability to process information about screening may be a key mediator in the relationship between socioeconomic status and screening participation [2,3]. Despite literacy levels being considered during the design phases of the current information booklet, it is still challenging to interpret, particularly for those with poor basic skills [4,5]. Research addressing inequalities in communication is needed if disparities in screening participation are to be ameliorated [6,7].

To address this issue we aimed to develop a ‘gist-based’ information leaflet that could supplement the existing information booklet ‘Bowel Cancer Screening: The Facts’. The leaflet is intended to be an additional, easy to read leaflet that provides essential information about CRC screening, without compromising the preferences of those that demand more detailed information [8].

### 1.2 Using theory and best practice guidelines to develop a supplementary leaflet

Best practice guidelines from the fields of information design, cognitive psychology and health literacy were used to complement a theory-based approach during the design phase [9–12]. To encourage informed decision-making, we ensured the leaflet met communication guidance from the European Union (EU) [13] and principles put forth by England's National Health Service (NHS) informed choice initiative [14]. As the leaflet was intended to supplement the existing information, the process of consent when making a screening decision is still met according to General Medical Council guidelines [15].

Fuzzy-trace theory (FTT) is a theory of judgement and decision making that has been applied to medicine and health [16]. It is a dual-processing theory which proposes that information is encoded into memory in two parallel forms: a ‘gist’ representation and a verbatim representation. Gist representations are vague, qualitative concepts that capture the ‘bottom-line’ meaning of information. As such, they are subjective to the individual and affected by a range of different core values, which themselves are influenced by factors such as emotional state, general world view and basic skill level. In contrast, verbatim representations are precise and quantitative, and capture the surface (or literal) form of information. Gist representations are formed along a continuum (analogous to scales of measurement), which range from the simplest to most complicated, i.e. categorical, ordinal and interval. Evidence shows that people (particularly older adults) have a consistent preference for using the simplest gist to make decisions [17–20].

Despite this preference, most official health information is presented in a verbatim format [17] and there is an increasing tendency to provide more information and choice to consumers in order to facilitate informed decision-making [21]. However, this tendency can have the unintended effect of interfering with decision-making processes; a so-called ‘more is less’ phenomenon [22–25]. Information should not be so oversimplified that it no longer allows informed decisions to be made [13,14], but presenting it in a format that is more closely aligned with preferred processing styles (i.e. gist) can reduce its cognitive burden [26], particularly for individuals with lower levels of literacy and numeracy [5,26]. This is because individuals with low basic skills often have difficulty in separating the relevant gist from non-essential information [23]. It is therefore recommended that gist-based information is presented separately to more detailed (verbatim) information [27]. The provision of a supplementary gist leaflet is therefore justified.

#### 1.2.1 Specific principles within the leaflet

Processing numerical information related to CRC screening was identified as a particular problem in our previous study of people reading existing information booklet supplied to individuals in the English CRC screening programme [4]. To overcome these difficulties, we attempted to encourage gist-based processing by providing a verbal description of the number which provides an evaluative label (i.e. gist) of the number (e.g. ‘most people [98 out of 100]’). This approach has been used successfully in previous research [28–30], with evidence to suggest it increases deliberative processing of the numerical information [31]. In line with current evidence, natural frequencies with the same denominator were used to present key numerical information [32].

In keeping with the ‘less is more’ approach [22], we further encouraged gist-based processing by removing specific concepts which were deemed ambiguous and non-essential in our previous study [4]. For example, when reading information about follow-up testing in the existing booklet, individuals responded with strong negative emotions which led to disengagement with the information. Text on this concept was therefore included, but it was kept to a minimum.

Additional literature was also consulted when identifying non-essential constructs. For example, the concept of preventing CRC was removed because of the unconvincing evidence that FOB-based screening reduces incidence of CRC [33]. We therefore focused on the primary mechanism by which FOB screening works; the early detection of colorectal adenomas. A further example of streamlining was the removal of academic references from within the text to accommodate the preferences of low literacy individuals [34]. The removal of non-essential concepts resulted in four pages of text being used for the gist leaflet, compared with 15 pages in ‘The Facts’ booklet.

Guidelines on the layout of health information designed for low literacy groups suggest providing essential information at the beginning of the text [9], as this has been shown to improve comprehension and decision-making [23]. We consulted experts in the field of cancer control to ascertain what should be considered essential information about the English CRC screening programme, and presented it first. We also provided clear sign-posting, including a directional prompt and written statements indicating where more detailed information could be found [35].

Health literacy, EU and NHS guidelines suggest vernacular rather than formal language should be used where possible in cancer communication materials [10,12–14]. These guidelines also recommend that information should be written in short sentences and bullet point lists. Evidence from cognitive psychology suggests this reduces the cognitive burden of information by enabling participants to ‘chunk’ information and retain more in short-term memory [36,37]. This is particularly important for individuals with poor basic skills due to the strong association between health literacy and cognitive ability [38].

The EU guidelines also suggest that the information materials should be appealing to the recipient [13]. In response to this, we chose to use a blue background because experimental evidence has demonstrated that it invokes a lower disgust response [39], a frequently cited barrier to CRC screening participation [40–42].

### 1.3 Aims

In line with a framework for the evaluation of patient information materials [43], we report on the readability and comprehensibility of the supplementary gist-based leaflet described above.

## 2 Methods

### 2.1 Participants

We recruited 28 participants via mail from two community organisations. Social Action for Health (SAfH) is a Non-Governmental Organisation (NGO) involved in health promotion within disadvantaged areas of London. ContinYou is an adult education organisation that works with children and adults in deprived communities. We also recruited participants from our Departmental research panel. Recruitment sites were specifically chosen in order to target and include the perspective of individuals who may struggle to access and use health information due to limited health literacy and numeracy skills. A number of barriers exist to the recruitment of such individuals, and we were mindful of these in our approach [44].

### 2.2 User-testing design

**Fig. 1:**
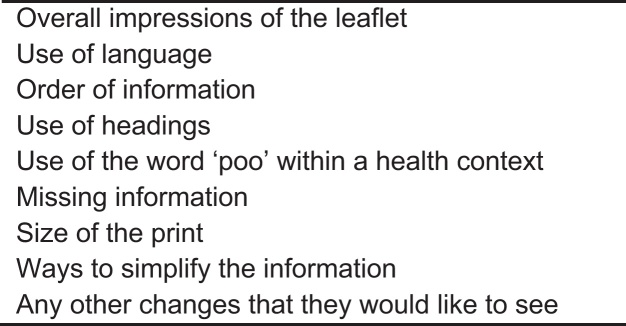
Qualitative interview topic guide.

We used a mixed-methods, user-testing approach to assess the comprehensibility of the information leaflet [45–47]. In rounds of approximately 8–10 people at a time, we identified problems with the gist-based leaflet. Both quantitative (face to face administered questionnaire) and qualitative (brief semi-structured interview) methods were used to achieve this purpose. Re-testing assessed the impact of revisions on a new set of participants, and was repeated as necessary (see Fig. 1).

Inclusion criteria were age 45–59 years (i.e. before the age at which CRC screening is offered in England) and no previous diagnosis of CRC. Exclusion criteria were not being able to speak or read English, previous CRC screening, and severe cognitive impairment. The study was approved by the UCL research ethics committee (Reference: 2247/002).

### 2.3 User-testing procedure

**Fig. 2:**
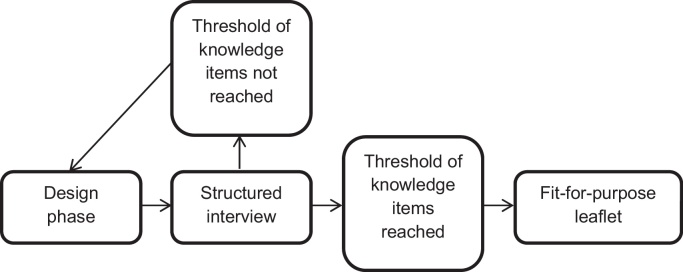
Procedure for user-testing a comprehensible leaflet.

Participants were asked to complete a brief socio-demographic questionnaire on arrival, followed by a health literacy assessment. They read through the gist-based leaflet for as long as they wanted, and completed a researcher-led comprehension test. The participant had access to the gist-based leaflet at all times. This was followed by a brief (5–10 min) semi-structured interview (see Fig. 2 for an overview of the topic guide).

### 2.4 Measures of participant characteristics

The following characteristics were recorded: age, gender, marital status (married/living with partner, single/divorced/separated, widowed), English as first language (yes/no), employment (currently employed, unemployed/disabled or too ill to work, retired), education level (basic high school qualifications or less [i.e. no formal qualifications, GCSEs or basic work qualifications], advanced high school qualifications or equivalent [i.e. A-levels or advanced work qualifications], university educated), health literacy (adequate, marginal/inadequate), experience with written documents (all the time, some of the time, hardly ever), previous cancer diagnosis (yes/no) and knowing someone else that has been diagnosed with cancer (yes/no).

Health literacy was assessed using the UK version of the Test of Functional Health Literacy in Adults (UK-TOFHLA) [48] which has numeracy and literacy sections. The numeracy section involves tasks relating to date and time calculation, computation of medication dosage, and patient navigation. This section takes approximately 10 min to complete. The literacy section is based on the ‘cloze’ procedure. Three passages of text (instructions on how to prepare for an X-ray, eligibility for NHS prescriptions and a consent form for surgery) of increasing difficulty are given to the participant and every fifth word is missing. Where a word is missing a blank line is drawn and 4 possible words that could be used are provided. This section takes approximately 12 min to complete. A score of 100 is calculated, with each section having a maximum score of 50. Scores are converted into three groups: inadequate (0–59), marginal (60–74), and adequate (75–100) health literacy [49].

### 2.5 Tested materials

The Flesch Kincaid formula [50] was used to calculate the reading ease of the gist-based leaflet. Scores range from 0 to 100, with higher scores indicating greater reading ease. The readability scores for version 1, 2 and 3 were 82.1, 79.4 and 81, respectively. This corresponded to a US grade level of 4–5 (equivalent to age 9–10 years). All versions of the gist-based leaflet that were tested can be found in the supplementary online material.

### 2.6 Outcome

The primary outcome was the percentage of participants correctly responding to eight true (T) or false (F) statements about CRC and CRC screening. In line with European guidelines for medicinal package testing [51], each statement had to be answered correctly by at least 80% of participants for our leaflet to be deemed legible, clear, and easy to read. The statements were based on the prevalence of CRC (1 statement), the logistics of the programme (4 statements), the potential for screening to reduce the likelihood of death from CRC (1 statement), and the risks of screening, including false-positives and false-negatives (2 statements). Measurement of these factors is in keeping with previous research that has assessed CRC screening knowledge [52] and the UK General Medical Council guidelines for consent [15]. The phrasing and response options mirrored the gist-based style of the leaflet [53,54].

### 2.7 Data analysis

We calculated the total number of individuals who answered each statement correctly (statement totals) as well as the mean number of statements correctly answered per participant (individual totals). Data from the semi-structured interviews were digitally recorded, transcribed verbatim, and analysed using thematic analysis, which is a qualitative technique for identifying patterns (themes) within data [55]. The purpose of the thematic analysis was to pin-point the particular areas of the gist-based leaflet that caused difficulties with comprehension.

## 3 Results

### 3.1 Participant characteristics

The majority of participants were female (75%), employed (54%), white (54%), had a GCSE level of education or below (57%), adequately literate (82%), without a partner (68%), spoke English as a first language (75%), and had either received a cancer diagnosis themselves (11%) or knew someone that had (82%). The majority had used written documents in their current of previous employment at least some of the time (75%) (see Table 1). As rounds progressed, more individuals had a lower level of education, marginal or inadequate health literacy scores, spoke English as a second language, or were from a minority ethnic group.

### 3.2 Round 1

#### 3.2.1 Round 1: quantitative findings

As demonstrated in Table 2, the majority of the statements were answered correctly by at least 80% of participants. However, two statements (‘The FOB test is done at home’ [T] and ‘People with an abnormal result always have cancer’ [F]), were answered correctly by less than 80% of participants. At an individual level, participants were able to answer a mean of 7.2 out of 8 statements correctly (range = 5–8).

#### 3.2.2 Round 1: changes to the leaflet

In response to the threshold not being met for the statement that ‘the FOB test is done at home’, we changed the word ‘post’ to ‘home’ in the following sentence to clarify where the test was completed: ‘A FOB test kit with instructions is sent through to the home’.

More than 20% of individuals did not correctly answer the statement that an abnormal test result does not necessarily mean cancer has been found. One participant commented that: ‘*I do wonder about the fact that if you have an abnormal test that it doesn’t necessarily indicate that you’ve got cancer. That's inferred but it doesn’t necessarily say that*’ (AL, 55 years, female, degree level education). To improve comprehension of the meaning of an abnormal result, we added the following sentence: ‘An abnormal result does not always mean cancer has been found’.

Our interviews demonstrated that the language used was easy to understand for the audience, ‘*It's quite well set out, and it's readable and gives you basically all the information*’ (WG, 58 years, female, no formal qualifications). However, further changes were identified by participants that could make it more accommodating for low literacy groups: ‘*There were a couple of words in it that I thought might need thinking about…‘discuss’, I wonder whether ‘talk about’ would be more appropriate?*’ (AL, 55 years, female, degree level education). Changes were also made to the spacing between and within lines to improve readability.

### 3.3 Round 2

#### 3.3.1 Round 2: quantitative findings

As demonstrated in Table 2, nearly all statements were answered correctly by at least 80% of the participants. However, the statement on the meaning of an abnormal result remained problematic (8. ‘People with an abnormal result always have cancer’ [F]). At a participant level, a mean of 7.1 out of 8 statements were answered correctly (range = 4–8).

#### 3.3.2 Round 2: changes to the leaflet

Changes to the layout of the leaflet were made in response to difficulties with remembering all of the information that they have just read, ‘*I think it's ok, but it's remembering what you read. If you read something and don’t remember, it doesn’t do you any benefit does it?*’ (DW, 52 years, female, no formal qualifications). Changes included placing boxes around text that related to each sub-heading, reducing the number of bullet points on the final page, changing the colour of the background and increasing the size of the font on the front page to increase the readability of the text for individuals with eyesight difficulties (‘*It's very clear. Maybe I would say, it could be done in more bigger letters, you know if somebody's old or something*’ (SF, 51 years, female, no formal qualifications)). These changes were particularly apparent on the final page which assisted participants when searching for the correct answer to the statement that did not meet the threshold. The text relating to this statement was altered: ‘For most people, the follow-up test will show there is no bowel cancer’ in an attempt to improve comprehension.

Participants reported being confused about the age of eligibility for screening: ‘That's all clear and it's explained further, all very simple. But this I couldn’t get [age extension]. That's like a random statement. It's not really backed up or [explained] why’ (VY, 45 years, male, advanced high school qualifications). Participants also wanted reassurance that the test was simple, as some felt that it might be complicated and that people may be less likely to participate as a result. This resulted in changes to the text concerning the age that people are invited to screening, as well as an additional sentence highlighting ‘The FOB test is easy to do’.

The title of the booklet (‘A two minute guide’) was changed as this may have been perceived as intimidating by less literate and slower readers: ‘*This is meant to be a two minute guide. Well people read at their own pace and you know they might think well, oh. A simple guide? Or is that being patronising…or the essentials?*’ (FV, 55 years, female, degree level education). Finally, the full title of the Faecal Occult Blood test was added in response to comments questioning the phrase, FOB test: ‘*I think the only thing is, FOB, what does that stand for?*’ (WF, 58 years, male, no formal qualifications).

### 3.4 Round 3

#### 3.4.1 Round 3: quantitative findings

As demonstrated in Table 2, all statements were answered correctly at least 80% of the time. The pre-defined threshold was therefore met and the leaflet was considered ‘fit-for purpose’. At a participant level, individuals were able to answer a mean of 7.2 out of 8 statements correctly (range = 6–8).

## 4 Discussion and conclusions

### 4.1 Discussion

The objectives of this study were to design and user-test a ‘gist-based’ colorectal cancer screening information leaflet, which promotes comprehension of the screening offer. Principles of Fuzzy Trace Theory complemented by best practice guidelines from the fields of information design, cognitive psychology and health literacy were used to provide accessible information about the aims, benefits and disadvantages of the English CRC screening programme. Readability scores indicated that the leaflet was suitable for individuals with low literacy (e.g. reading age: 9–10 years), and may therefore increase the accessibility of the programme to disadvantaged groups. User-testing indicated that the leaflet was well comprehended in all rounds and after three rounds of testing, the pre-defined threshold was reached.

In round 1, two statements did not meet the comprehension threshold. These related to where screening takes place and the meaning of an abnormal result. This finding was supported by qualitative data, which also highlighted additional text that could be simplified. Changes were made to the content of the leaflet and an additional round of testing was performed. In round 2, responses to the abnormal result item were still not adequate. In this round, qualitative comments focussed on the design and layout of the text. Changes made to the final version of the gist leaflet encouraged readers to ‘chunk’ information and made differences between sections more concrete. This reduced the cognitive load of the text, which may be a barrier to information processing among disadvantaged groups [36,37]. In the third round of testing, the pre-defined threshold was met and the leaflet was considered fit-for-purpose.

### 4.2 Strengths and limitations

A strength of this research was the theoretical underpinning and the use of best practice guidelines from a number of different fields. FTT has been widely discussed in the literature over the last two decades [16,56], however, there have been few reports of public health interventions that have tested its hypotheses. Here, we demonstrate how gist-based information could be operationalised within the constraints of an organised healthcare system. Furthermore, while this leaflet was intended to supplement existing information materials and not act as an independent decision aid, it was reassuring that it met International Patient Decision Aids Standards (IPDAS) criteria for the design of decision aids for low literacy groups. These criteria are that the leaflet is easily understandable by the target group and should have a readability of a grade 8 or equivalent [9].

The sample reported here were less literate or educated than national estimates [48,57] and the inclusion of such groups within the initial stages of intervention design is recommended [58]. However, the majority of print and multimedia interventions fail to report on how they involved the target populations in their development [59], despite their inclusion mitigating socioeconomic differences in response to public health interventions [60]. Nonetheless, the study may have benefited from the inclusion of more low literacy individuals. This is demonstrated by the observation that several participants had a degree level education and they contributed disproportionately to the discussion.

An implication of the relatively literate sample is that the gist leaflet may not have addressed the concerns of those most in need of supplementary communication materials. Furthermore, the number of correct responses to the comprehension questions may have been lower if a sample of individuals with lower levels of literacy had participated. This would have resulted in more rounds of testing and more changes being made to its current design. Future research should focus not only on the recruitment of low literacy groups, but also on ways to promote their engagement with the research process once they have consented. For example, using lay members of the community to chair focus groups, improving research instructions so that they are easily comprehendible and ensuring participants’ continued involvement throughout the research process, are some possibilities.

Small sample sizes are the norm in user-testing studies, but chance variation between individuals means that the results may be less generalisable to the wider population. Although the methodology allows us to observe levels of comprehension, it does not consider the wider determinants of screening behaviour [2]. In addition, because of the length of the user-testing task and literacy assessments, we did not ask respondents to elaborate on their open-ended statements. As such, the data were often brief utterances rather than in-depth comments. These limitations will be addressed in our future research plans, which will test the communicative effectiveness of the leaflet [43] in larger, more generalisable populations.

### 4.3 Conclusion

In conclusion, we have shown that it is possible to use FTT as a guiding framework to design gist-based CRC screening information that is comprehensible to all literacy groups. Best practice guidelines were useful supplements to this theory-driven process and they provided explicit guidance on how to address comprehension difficulties specific to low literacy groups. Further testing of the leaflet is now required to assess its communicative effectiveness.

### 4.4 Practical implications

To our knowledge, this report is the first application of a user-testing methodology in the cancer control context. A similar methodology could be used to assess comprehension of other cancer communication interventions including multimedia resources, online information and patient–physician communication.

User-testing improved the communicative effectiveness of the supplementary gist-based information leaflet. It will now be evaluated as part of a large national randomised controlled trial designed to reduce socioeconomic inequalities in CRC screening participation.

## Figures and Tables

**Table 1:** Participant characteristics in user-testing.

	Round 1 (*n* = 6)	Round 2 (*n* = 11)	Round 3 (*n* = 11)	Total (*n* = 28)
Gender				
Male	2 (33)	4 (36)	1 (9)	7 (25)
Female	4 (67)	7 (64)	10 (91)	21 (75)
Marital status				
Married/living with partner	1 (17)	5 (45)	3 (27)	9 (32)
Single/divorced/separated	5 (83)	5 (45)	7 (64)	17 (61)
Widowed	0 (0)	1 (9)	1 (9)	2 (7)
English as first language				
Yes	6 (100)	7 (64)	8 (73)	21 (75)
No	0 (0)	4 (36)	3 (27)	7 (25)
Employment				
Currently employed	2 (33)	7 (64)	6 (55)	15 (54)
Unemployed/disabled or too ill to work	3 (50)	4 (36)	5 (45)	12 (43)
Retired	1 (17)	0 (0)	0 (0)	1 (4)
Education				
≤Basic high school qualifications	5 (83)	4 (36)	7 (64)	16 (57)
Advanced high school qualifications or equivalent	0 (0)	4 (36)	1 (9)	5 (18)
University educated	1 (17)	3 (27)	3 (27)	7 (25)
Health literacy^a^				
Adequate	6 (100)	9 (82)	8 (73)	23 (82)
Marginal/inadequate	0 (0)	1 (9)	3 (27)	4 (14)
Ethnicity				
White	6 (100)	4 (36)	5 (45)	15 (54)
Non-White	0 (0)	7 (64)	6 (55)	13 (46)
Use of written documents				
All or most of the time	1 (17)	7 (64)	3 (27)	11 (39)
Some of the time	3 (50)	2 (18)	5 (45)	10 (36)
Hardly ever	2 (33)	2 (18)	3 (27)	7 (25)
Previous cancer diagnosis				
Yes	1 (17)	0 (0)	2 (18)	3 (11)
No	5 (83)	11 (100)	9 (82)	25 (89)
Know at least one person diagnosed with cancer				
Yes	5 (83)	8 (73)	10 (91)	23 (82)
No	1 (17)	3 (27)	1 (9)	5 (18)

a One participant refused to complete the TOFHLA health literacy assessment in round 2. % is reported for the total number of participants in this round. The total % also includes this individual.

**Table 2:** Participant responses in rounds 1, 2 and 3.

	Round		
	1	2	3
	Correct *n* (%)	Correct *n* (%)	Correct *n* (%)
1. Doing the FOB test lowers the risk of dying from bowel cancer [T]	6 (100)	11 (100)	11 (100)
2. The FOB test is done at home [T]	4 (67)	10 (91)	9 (82)
3. Most people who do the FOB test will receive an abnormal result [F]	5 (83)	9 (82)	9 (82)
4. Only women are sent a FOB test [F]	6 (100)	11 (100)	11 (100)
5. Bowel cancer is a common cancer in people over 60 [T]	6 (100)	10 (91)	10 (91)
6. People only need to do the FOB test once in their life [F]	6 (100)	10 (91)	11 (100)
7. The FOB test can miss bowel cancer [T]	6 (100)	9 (82)	9 (82)
8. People with an abnormal result always have cancer [F]	4 (67)	8 (73)	9 (82)
