# Supplementary material for: The development and testing of a brief (‘gist-based’) supplementary colorectal cancer screening information leaflet
Source: Patient Educ Couns. 2013 Dec;93(3):619–25. doi: 10.1016/j.pec.2013.08.013 (PMC3863947; doi:10.1016/j.pec.2013.08.013)
Supplement: Supplementary file 1 [file mmc1.pdf]

*There is more information about the FOB test in the leaflet, 'Bowel Cancer Screening: The Facts'*

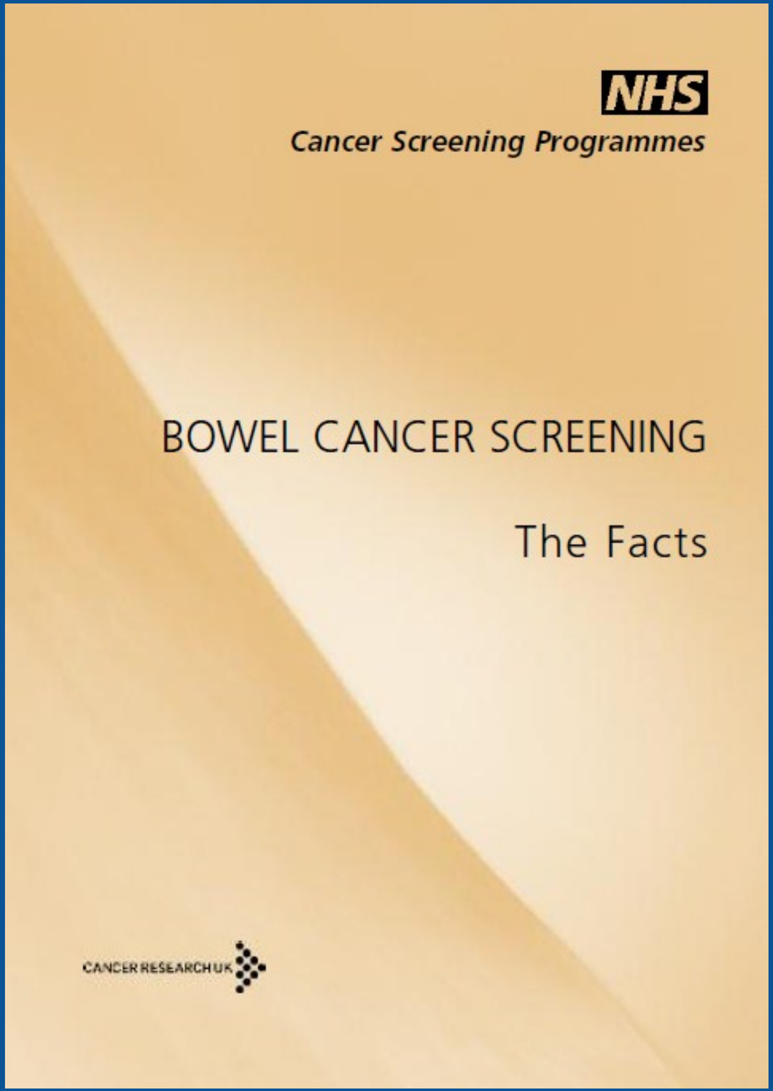


# Bowel Cancer Screening: A Short Guide

## *The essentials:*

- Bowel cancer is a common cancer in people 60 and over
- A screening test (called the FOB test) can spot signs of bowel cancer early
- Doing the FOB test lowers the risk of dying from bowel cancer
- Everyone aged 60-69 is sent the FOB test to do at home every two years
- In the future, kits may be sent to people 70 and over too

*If you would like to know more about the FOB test, see inside for details*

## How does the FOB test work?

- The FOB test checks for tiny amounts of blood in stools (poo) that cannot be seen by the eye
- Blood in stools can be a sign of bowel cancer
- A FOB test kit with instructions is sent through the post
- The FOB test is done at home by putting small amounts of stool onto the test kit
- The test kit is sent back to a laboratory in a freepost envelope

## What happens after the FOB test is done?

- The FOB test result is sent to the home within two weeks
- Most people (98 out of 100) have a normal result
- People with a normal result will be automatically sent another FOB test kit every two years until they are 70
- A small number of people (2 out of 100) have an abnormal result
- People with an abnormal result are offered an appointment to discuss further testing

## How accurate is the FOB test?

- The FOB test lowers the risk of dying from bowel cancer
- Like all screening tests, the FOB test is not 100% accurate
- Bowel cancer that is not bleeding at the time of testing can be missed
