# Supplementary material for: The development and testing of a brief (‘gist-based’) supplementary colorectal cancer screening information leaflet
Source: Patient Educ Couns. 2013 Dec;93(3):619–25. doi: 10.1016/j.pec.2013.08.013 (PMC3863947; doi:10.1016/j.pec.2013.08.013)
Supplement: Supplementary file 2 [file mmc2.pdf]

For more information see the enclosed leaflet: *'Bowel Cancer Screening: The Facts'*

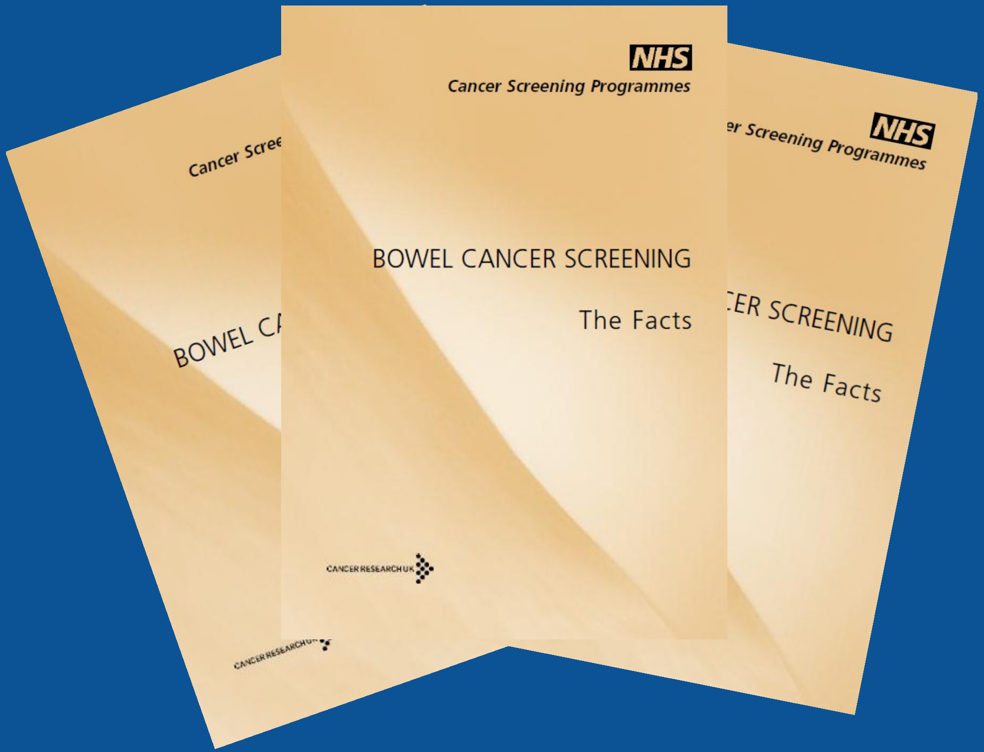

The NHS Bowel Cancer Screening Programme: A Two Minute Guide

The essentials:

- Bowel cancer is a common cancer in people aged 60 and over
- A screening test (called the FOB test) can spot signs of bowel cancer early
- Bowel cancer screening is meant for men and women, even if they do not have symptoms or bowel problems
- Doing the FOB test has been proven to lower the risk of dying from bowel cancer
- Everyone aged 60-69 is sent the FOB test to do at home every 2 years
- The screening programme is starting to send kits to people aged 70-74

If you would like to know more about the FOB test, see inside for details

How does the FOB test work?

- The FOB test checks for tiny amounts of blood in stools (poo) that cannot be seen by the eye
- Blood in stools can be a sign of bowel cancer
- A FOB test kit with instructions is sent through to the home
- The FOB test is done at home by putting small amounts of stool onto the test kit
- The test kit is sent back to a laboratory in a freepost envelope

What happens after the FOB test is done?

- The FOB test result is sent to the home within two weeks
- Most people (98 out of 100) have a normal result
- People with a normal result will automatically be sent another FOB test kit every two years until they are 70
- A small number of people (2 out of 100) have an abnormal result
- An abnormal result does not always mean cancer has been found
- People with an abnormal result are offered an appointment with a specialist to talk about further testing

How accurate is the FOB test?

- The FOB test lowers the risk of dying from bowel cancer
- Like all screening tests, the FOB test is not 100% accurate
- Bowel cancer that is not bleeding at the time of testing can be missed

To find out where you can get more information, see the back page
