# Supplementary material for: The development and testing of a brief (‘gist-based’) supplementary colorectal cancer screening information leaflet
Source: Patient Educ Couns. 2013 Dec;93(3):619–25. doi: 10.1016/j.pec.2013.08.013 (PMC3863947; doi:10.1016/j.pec.2013.08.013)

NHS Bowel Cancer Screening Programme: The essentials

- Bowel cancer is a common cancer in people aged 60 and over
- The Faecal Occult Blood (FOB) test can spot signs of bowel cancer early
- The FOB test is for men and women, even if they do not have bowel problems
- Doing the FOB test lowers the risk of dying from bowel cancer
- Everyone aged 60-69 is sent the FOB test to do at home every 2 years

If you would like to know more about the FOB test, see inside for details

DEVELOPED BY UNIVERSITY COLLEGE LONDON WITH FUNDING FROM THE NATIONAL INSTITUTE FOR HEALTH RESEARCH

NHS
National Institute for Health Research

How does the FOB test work?

- The FOB test checks for tiny amounts of blood in stools (poo) that cannot be seen by the eye
- Blood in stools can be a sign of bowel cancer
- An FOB test kit with simple instructions is sent to your home
- The FOB test is easy to do
- You do the FOB test at home by putting small amounts of stool onto a test kit
- You can send the test kit back to the laboratory in a special freepost envelope

What happens after you've done the FOB test?

- Everyone gets their FOB test result through the post within two weeks
- Most people (98 out of 100) get a normal result
- People with a normal result will be sent another FOB test every 2 years up to age 69
- A small number of people (2 out of 100) get an abnormal result
- People with an abnormal result are offered an appointment at a screening centre to talk about further testing
- For most people, the follow up test will show there is no bowel cancer

How accurate is the FOB test?

- Doing the FOB test lowers the risk of dying from bowel cancer
- Like all screening tests, the FOB test is not 100% accurate
- Bowel cancer can be missed if there is no bleeding at the time the FOB test is done

For more information see the enclosed leaflet: *'Bowel Cancer Screening: The Facts'*

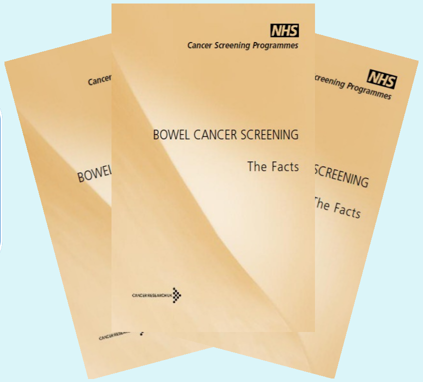

Supplement: Supplementary file 3 [file mmc3.pdf]
